# Disruption of Membranes of Extracellular Vesicles Is Necessary for ELISA Determination of Urine AQP2: Proof of Disruption and Epitopes of AQP2 Antibodies

**DOI:** 10.3390/ijms17101634

**Published:** 2016-09-26

**Authors:** Masaaki Nameta, Yoko Saijo, Yasukazu Ohmoto, Kiyonori Katsuragi, Keiko Yamamoto, Tadashi Yamamoto, Kenichi Ishibashi, Sei Sasaki

**Affiliations:** 1 Electron Microscope Core Facility, Niigata University, Niigata 951-851, Japan; nametan@med.niigata-u.ac.jp; 2 Department of Research and Development, Diagnostic Division, Otsuka Pharmaceutical Co., Ltd., Tokusima 771-0182, Japan; saijoy@otsuka.jp (Y.S.); katsuragik@otsuka.jp (K.K.); 3 Department of Medical Innovations, New Drug Research Division, Otsuka Pharmaceutical Co., Ltd., Tokusima 771-0192, Japan; ohmoto.yasukazu@otsuka.jp; 4 Biofluid Biomarker Center, Institute of Social Innovation and Promotion, Niigata University, Niigata 950-2181, Japan; keikoy-bbc@ccr.niigata-u.ac.jp (K.Y.); tadashiy-bbc@ccr.niigata-u.ac.jp (T.Y.); 5 Department of Pathophysiology, Meiji Pharmaceutical University, Tokyo 204-8588, Japan; kishiba@my-pharm.ac.jp; 6 Department of Nephrology, Tokyo Medical and Dental University, Tokyo 113-8519, Japan

**Keywords:** aquaporin-2 (AQP2), exosome, extracellular vesicle, ELISA, biomarker, kidney, water-balance

## Abstract

Aquaporin-2 (AQP2) is present in urine extracellular vesicles (EVs) and is a useful biomarker for water balance disorders. We previously found that pre-treatment of urine with alkali/detergent or storage at −25 °C is required for enzyme-linked immunosorbent assay (ELISA) measurement. We speculated that disruptions of EVs membranes are necessary to allow for the direct contact of antibodies with their epitopes. Human urine EVs were prepared using an ultracentrifugation method. Urine EV samples were stored at different temperatures for a week. Electron microscopy showed abundant EVs with diameters of 20–100 nm, consistent with those of exosomes, in normal urine, whereas samples from alkali/detergent pre-treated urine showed fewer EVs with large swollen shapes and frequent membrane disruptions. The abundance and structures of EVs were maintained during storage at −80 °C, but were severely damaged at −25 °C. Binding and competitive inhibition assays showed that epitopes of monoclonal antibody and polyclonal antibody were the hydrophilic Loop D and C-terminus of AQP2, respectively, both of which are present on the inner surface of EVs. Thus, urine storage at −25 °C or pre-treatment with alkali/detergent disrupt EVs membranes and allow AQP2 antibodies to bind to their epitopes located inside EVs.

## 1. Introduction

Aquaporin-2 (AQP2) is a key molecule that determines the urine concentrating ability of the kidneys [1,2,3]. AQP2 is excreted in the urine, and most urinary AQP2 is embedded in membranes of extracellular vesicles (EVs) [4,5,6]. Urine AQP2 may serve as a biomarker for water balance disorders, including congestive heart failure and liver cirrhosis [7,8,9,10]. In a previous study [11], we found that urine samples stored at 4 °C or −80 °C did not show significant AQP2 values, whereas those stored at −25 °C showed stable values in enzyme-linked immunosorbent assay (ELISA) measurement. Moreover, urine samples treated with alkali/detergent showed consistent and comparable values to those obtained from urine stored at −25 °C. These observations may be explained by the following: (1) the epitopes of AQP2 antibodies (recognition sites) within the AQP2 molecule face the inside of EVs; (2) EV membranes are resistant to breakage, preventing direct contact of antibodies with their epitopes; and (3) storage at −25 °C or alkali/detergent pretreatment cause breaks in EV membranes. However, these hypotheses have not been verified. In this study, we evaluated the localization of antibody epitopes and observed the changes in the structures of EVs with an electron microscope (EM).

## 2. Results

### 2.1. Electron Microscope (EM) Observation 

**Figure 1:**
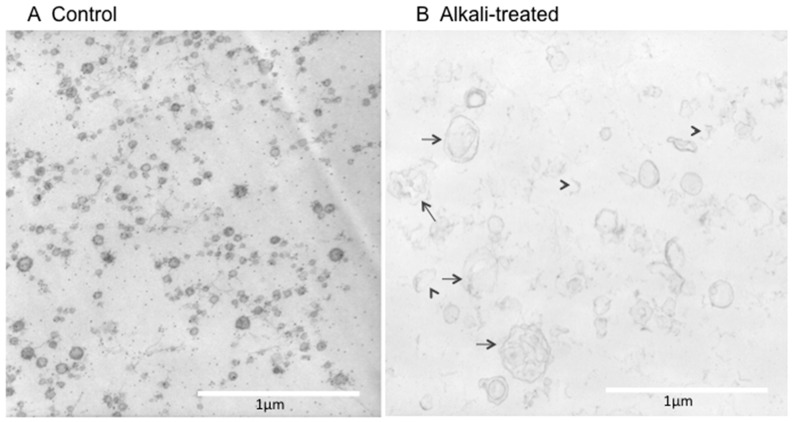
Electron microscope (EM) observation of extracellular vesicles (EVs). (**A**) Control: EV samples obtained from normal urine showed abundant 20–100-nm vesicles; (**B**) Alkali-treated: Urine samples were pre-treated with 0.4 N of NaOH and 0.5% Triton X-305 for 20 min followed by neutralization with HCl, and EVs were obtained in a similar manner. Disruption of EV membranes (arrow heads) and large vesicles composed of fused vesicles (arrows) were observed.

Urine EV samples were prepared from untreated human urine or urine treated with alkali/detergent for 20 min by an ultracentrifugation (see Materials and Methods). As shown in Figure 1A, vesicles with diameters of 20–100 nm were abundantly observed in normal urine. The size and shape of these vesicular structures were consistent with those of exosomes [6,12]. In contrast, EVs obtained from alkali/detergent-treated urine showed larger swollen vesicles (Figure 1B). The density of these vesicles membranes appeared to be thin, and many were disrupted (arrow heads, Figure 1B). Some of the vesicles became larger, possibly by fusing each other (arrows, Figure 1B). Similar observations were made in three independent experiments.

**Figure 2:**
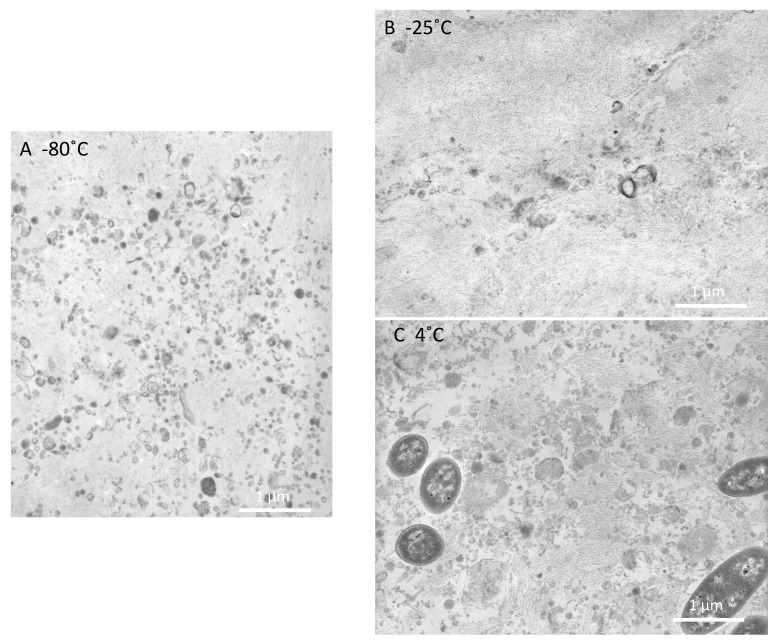
Effects of storage temperatures on EV structures. EV samples were stored at 4, −25, or −80 °C for 1 week, and prepared for EM observation. (**A**) Abundant small vesicles were observed in the specimen stored at −80 °C; (**B**) few vesicle structures were observed in the specimens stored at −25 °C; (**C**) the specimens stored at 4 °C showed fewer vesicles, some of which appeared to be enlarged.

To examine the effects of storage temperatures on the structure of EVs, EV samples were stored at 4, −25, and −80 °C for one week. Abundant small vesicles were observed in the specimen stored at −80 °C (Figure 2A), and the sizes were the same as those observed in fresh urine (Figure 1A). In contrast, vesicle structures were rarely observed in specimens stored at −25 °C (Figure 2B). The specimens stored at 4 °C showed fewer vesicles with a slightly swollen appearance (Figure 2C). This sample contained fragments of bacteria that may have grown during storage at 4 °C. Similar findings were observed in three separate studies. These observations are consistent with our previous ELISA measurements in that AQP2 values were only detectable when samples were stored −25 °C or pre-treated with alkali/detergent and were not detectable when stored at 4 °C or −80 °C.

### 2.2. Epitopes of the AQP2 Antibodies 

**Figure 3:**
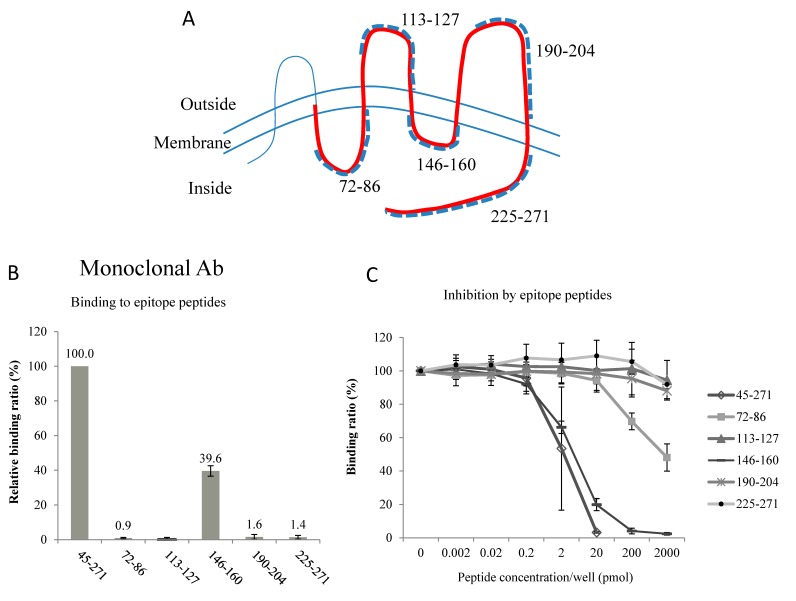
Determination of epitopes of the monoclonal antibody. (**A**) Topology of aquaporin-2 (AQP2) molecule with six transmembrane domains with N- and C-terminus inside the cell. Bold red line indicates the immunogen sequence (45–271) and dotted bold lines are synthetic peptides used for binding assay (**B**) and competitive inhibition assay (**C**).

Regarding the monoclonal antibody, the binding assay showed that this antibody had affinity to the peptide 146–160 as well as to the immunogen peptide 45–271 (Figure 3B). The binding to the peptide 146–160 was 39.6% ± 3.0% of the binding to the immunogen 45–271 (mean ± standard deviation (SD), *n* = 3). No significant binding to other peptides was detected. The inhibition assay also showed that binding of this antibody to the peptide 45–271 was inhibited by peptide 146–160 as well as peptide 45–271 (Figure 3C). At the peptide concentration of 20 pmol/well, the inhibition was 80.1% ± 3.6% (mean ± SD, *n* = 3), indicating a strong inhibition.

**Figure 4:**
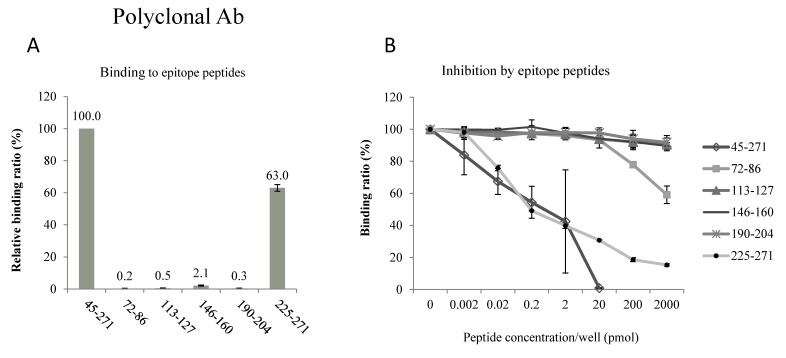
Determination of epitopes of the polyclonal antibody. Five synthetic peptides were used for binding assay (**A**) and competitive inhibition assay (**B**).

Similar studies were performed for the epitope of the polyclonal antibody. This antibody selectively bound to peptide 225–271 (63.0% ± 2.1%, *n* = 3, Figure 4A), and its binding to the immunogen peptide 45–271 was competitively blocked by this peptide (mean ± SD, *n* = 3, Figure 4B). 

These results indicate that the epitopes of these 2 antibodies face the intracellular side of the AQP2 molecule. Because the orientation of membrane proteins in EV membranes is the same as in cells (intracellular = intravesicular) [12], both of our antibodies recognized the intravesicular side of the AQP2 molecule. Thus, the disruption of EV membranes is necessary for antigen-antibody binding.

## 3. Discussion

Alkali/detergent pre-treatment and storage at −25 °C disrupted EV membranes (Figure 1 and Figure 2), supporting our previous hypothesis. The importance of the orientation of the antibody epitopes, i.e., whether they face inside or outside of vesicles, has not been thoroughly examined [13]. Recently, we [11] and Salih et al. [14] found that disruption/lyses of EV membranes was required for ELISA measurements of urine AQP2 and the Na-Cl cotransporter, because the epitopes for the antibodies are inside EVs. Accordingly, we conducted the alkali/detergent treatment (0.4 N of NaOH for 20 min together with 0.5% Triton X-305), whereas Salih et al. used 0.01% sodium dodecyl sulfate (SDS) for 10 min to disrupt EV membranes [11,14]. In our experience, the efficacy of vesicle lyses was more prominent following alkali/detergent treatment compared with after treatment with other detergents alone, although more thorough studies are necessary to confirm this [11]. The implication of this fact is important. Many studies have been conducted in which urine AQP2 values were immunologically measured via ELISA or radioimmunoassay. Those studies may have missed a considerable amount of urine AQP2. Currently, methods for disrupting EV membranes have been adopted in several studies involving ELISA for urinary AQP2 measurements [10,15]. The localization of the antibody epitope is not unique to AQP2, but also applies to other AQPs where the antibody epitopes are typically at the C-terminus, which are located inside EVs.

Notably, the structures of EVs stored at −25 °C were severely disrupted compared to those stored at −80 °C. The mechanism by which storage at −25 °C causes breakage of the EV membranes remains unknown. Fluctuation of membrane fluidity of EV membranes at −25 °C may cause membranes breakage. Thus, EV samples or urine samples should be stored at −80 °C and not at −25 °C.

The epitopes of antibodies that were raised against the recombinant 45–271 polypeptide are located on the intracellular domains of AQP2. The C-terminus has been shown to be a good region for raising high-quality antibodies, as was the case for our polyclonal antibody. We did not expect to find that Loop D was the epitope of our monoclonal antibody. The hydrophilic Loop D connects transmembrane domains IV and V; this region is not frequently used to raise antibodies, and its physiological significance remains unknown. However, a recent X-ray structure analysis clearly showed that Loop D interacts with the C-terminus of AQP2 on the cytoplasmic surface, facilitating conformational changes of the C-terminus, which are important for the intracellular signaling and trafficking of AQP2 [16]. Thus, Loop D is another important target for developing new drugs to regulate body water balance. Nevertheless, using two different antibodies with different epitopes could increase the specificity and sensitivity of our ELISA methods [11,17].

## 4. Materials and Methods 

### 4.1. EM

Human urine EV samples were prepared from fresh urine or those treated with alkali/detergent. In the alkali/detergent treatment, urine was alkalized by NaOH (final 0.4 N) together with 0.5% Triton X-305 for 20 min, followed by neutralization with HCl [11]. EVs were prepared by an ultracentrifugation method [18]. In brief, urine samples were centrifuged at 3000× *g* for 15 min to remove sediments, cells, cell debris, and others. Then, the supernatant was centrifuged at 17,000× *g* for 15 min to remove heavier membrane fractions. The supernatant was ultracentrifuged at 200,000× *g* for 1 h, and the pellet was suspended in phosphate buffered saline (PBS) or used for EM specimen preparation.

The pellets obtained after ultracentrifugation at 200,000× *g* were pre-fixed with a 2.5% glutalaldehide PBS solution followed by embedding in 1% agarose gel. The specimens were post-fixed in 1% osmium acid, dehydrated in ethanol, and embedded in Epok812. Ultrathin sections were prepared and contrasted with uranyl acetate and lead citrate for observations under a Hitachi H-600A electron microscope (Hitachi High-Technologies Corporation, Tokyo, Japan). 

### 4.2. Epitopes of the Antibodies

In our developed AQP2 ELISA method, we used two different anti-AQP2 antibodies to perform a sandwich ELISA. The immunogen was a recombinant human AQP2 protein corresponding to amino acids 45–271 of the AQP2 molecule (see Figure 3A), and a mouse monoclonal antibody and a rabbit polyclonal antibody were raised [17]. The mouse monoclonal antibody was used to coat the wells of the plate for capturing AQP2, and the rabbit polyclonal antibody was used to enhance the sensitivity and specificity (sandwich ELISA). In the present study, the epitopes of these antibodies were determined using two methods. In the binding assay, 6 different peptides corresponding to hydrophilic loop regions of AQP2 (see the topology of AQP2 in Figure 3A) were coated on the bottom of 96-well plates and diluted antibodies were added to wells. The bound antibodies were detected using horseradish peroxidase (HRP)-conjugated anti-mouse IgG antibody (Invitrogen Corporation, Camarillo, CA, USA). Each value was a mean of three measurements, and three separate sets of studies were performed. In the inhibition assay, antibodies were pre-incubated with the peptides, and the mixture was then added to wells coated with the 45–271 AQP2 recombinant protein. After incubation for 2 h, the plate was washed and the bound antibodies were detected using HRP-conjugated anti-IgG antibodies (Invitrogen). Each value was a mean of two measurements, and three separate sets of studies were performed. Data are shown as mean ± SD.

Human urine samples were obtained from healthy subjects who did not have recent kidney or urinary tract diseases. All participants provided written informed consent, and the study was conducted in accordance with Declaration of Helsinki. The protocol was approved by the ethical committee of Meiji Pharmaceutical University (No. 2609, Tokyo, Japan). 

## 5. Conclusions

In this study, we directly observed the disruption of EVs membranes when urine was stored at −25 °C or pre-treated with alkali/detergent and showed the inside vesicular localization of the epitopes of AQP2 antibodies, confirming our previous speculation for ELISA measurements of urine AQP2. The orientation of the antibody epitopes, i.e., whether they face inside or outside of vesicles is critically important in immunologic determination (ELISA) of membrane proteins contained in EVs.
